# Analysis of Dendritic Specializations in Two Classes of Kenyon Cells in the Mushroom Body of the Adult Honeybee, *Apis mellifera*

**DOI:** 10.1002/cne.70169

**Published:** 2026-05-18

**Authors:** Andrea Rafaela Nicolaidou, Basil el Jundi, Wolfgang Rössler, Claudia Groh

**Affiliations:** ^1^ Behavioral Physiology and Sociobiology, Biocenter University of Würzburg Würzburg Germany; ^2^ Institute of Biology and Environmental Sciences University of Oldenburg Oldenburg Germany

**Keywords:** dendritic claws, dendritic spines, microglomeruli, mushroom body calyx, olfaction, projection neurons, vision

## Abstract

The mushroom bodies (MBs) in the insect brain serve sensory integration and memory formation. In the honeybee, they house two classes of intrinsic neurons: class I (spiny) and II (clawed) Kenyon cells (KCs). Both classes form postsynaptic elements in synaptic complexes (microglomeruli) comprising large axonal boutons from olfactory and visual projection neurons. To adapt their neuronal information processing systems, MB microglomeruli undergo age‐, memory‐, and environment‐related structural plasticity. To analyze KC dendritic specializations and their connections with presynaptic boutons, we combined tracer injections in small groups of KCs from different age cohorts (freshly emerged bees to foragers) with presynaptic anti‐synapsin immunolabeling. Using high‐resolution confocal 3D reconstructions, we analyzed shape and contacts of class I and II KC dendrites in the olfactory (lip) and visual (collar) input sites of the MB calyx. In both KC classes, dendrites are always restricted to either the lip or the collar. We classified two types of class II KCs regarding the spatial distribution of dendritic branches: large‐clustered and small‐distributed. Individual claws of class II KCs largely vary regarding surface areas covered on individual axonal boutons (∼5%–70%). In class I KCs, we found four distinct morphological spine categories: stubby, thin, mushroom‐shaped, and branched. Interestingly, the overall frequency of putative spine–bouton contacts in class I KCs remains largely constant throughout age cohorts. We discuss the results in the light of structural dynamics in MB microglomerular circuits and their role in multisensory information processing.

## Graphical abstract

Honeybee mushroom bodies (MBs), sites for learning and memory, house two classes of intrinsic neurons: class I and II Kenyon cells (KCs) that form synaptic complexes with boutons of projection neurons (PNs) from primary sensory neuropils. Dendrites of both KC classes were restricted to distinct unimodal MB compartments. Dendritic spines of class I KCs could be subdivided into four categories, while class II KC dendrites could be classified into two distinct morphological subtypes. Spine–bouton frequencies remained constant throughout age cohorts.

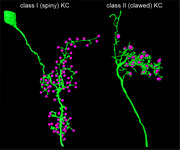

## 1 Introduction

Both aging and associated changes in the environment animals experience may strongly affect the nervous system all the way through adulthood. This has potential implications for neuronal maturation, sensory processing, and learning and memory in a wide range of species. To gain an understanding of the ability of the adult nervous system to form, maintain, and reorganize neuronal connections, detailed knowledge of the connectivity within neuronal microcircuits is necessary (for reviews, see Bailey and Kandel 1993; Kolb and Whishaw 1998; Tavosanis 2012). In the present study, we used the European honeybee *Apis mellifera* as a favorable experimental model to characterize structural neuronal circuit organization and its plasticity. We carried out high‐resolution analyses of putative anatomical connections between large presynaptic boutons of cholinergic olfactory and visual projection neurons (PNs) and dendritic specializations of postsynaptic neurons within main sensory input regions of a high‐order brain center, the mushroom body (MB).

**FIGURE 1:**
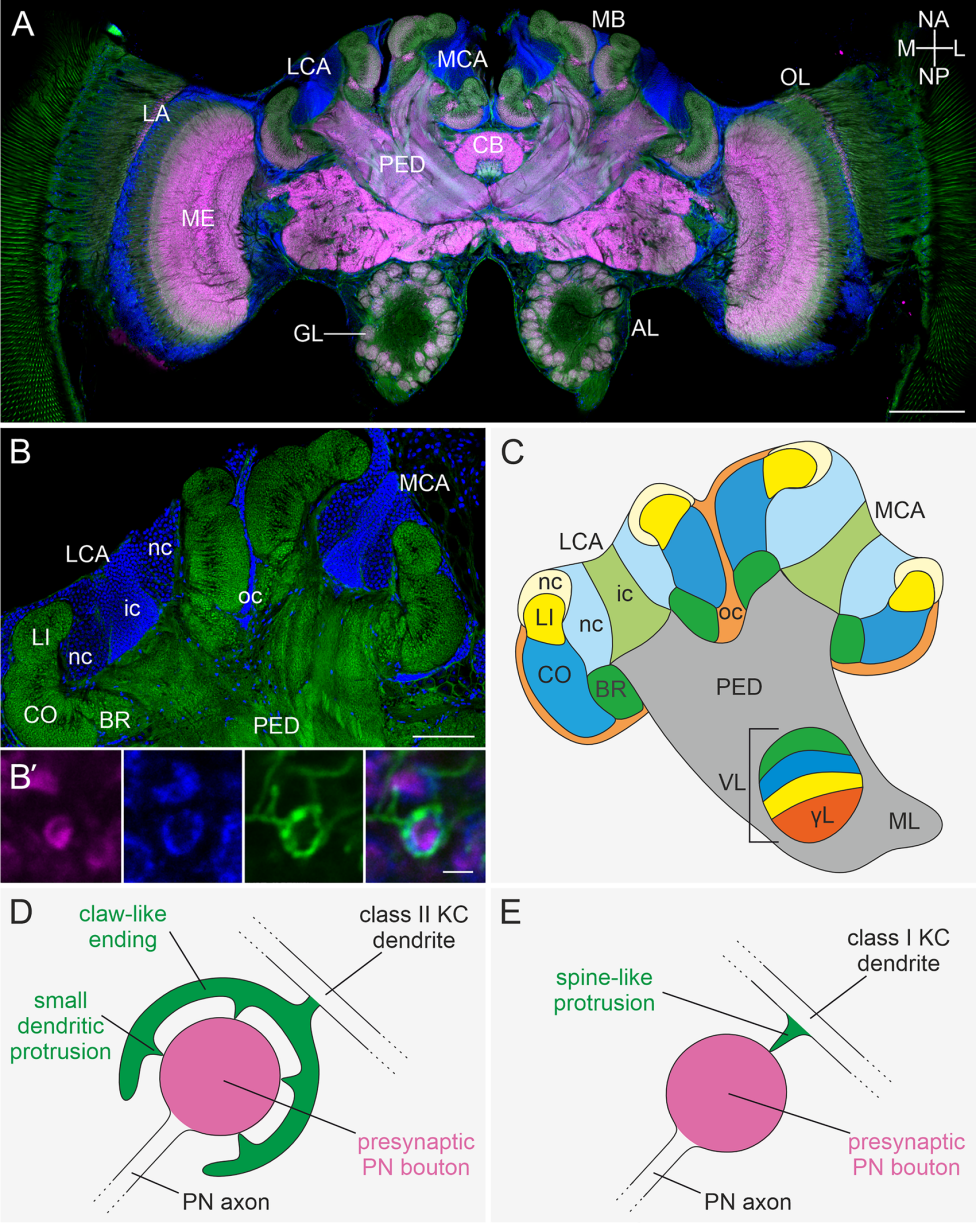
Anatomical organization of the MBs of an adult worker bee. (A) Frontal view of a central plane in the brain labeled with Hoechst (blue), anti‐synapsin (magenta), and f‐actin–phalloidin (green), showing the major neuropils and cell body clusters. Neuraxes: L, lateral; M, medial; NA, anterior; NP, posterior. (B) Higher magnification of MB calyces (BR, basal ring; CO, collar; LI, lip) and peduncle (PED) labeled with f‐actin–phalloidin (green). Hoechst labeling (blue) visualizes the KC nuclei (somata) arrangement clustered in inner compact (ic), noncompact (nc), and outer compact (oc) cells. (B’) High magnification of the modular synaptic layout of the calyx. Synapsin‐positive PN boutons (magenta) are encircled by f‐actin‐rich postsynaptic profiles (blue) and micro‐Ruby‐traced KC claws (green), forming MG (overlay). Main dendritic branches are devoid of phalloidin labeling. (C) Schematic ground plan of the honeybee MB. Modality‐specific input from class I spiny KCs from the LI, CO, and BR is maintained in distinct divisions in the vertical lobe (VL) above the layer of the gamma lobe (γL). The medial lobe (ML) extends toward the brain's midline. KC populations are color‐coded corresponding with the calycal region they supply; oc (clawed, class II) KCs are colored in orange. (D, E) Organization of an individual MG. Schematic illustration of a single PN bouton innervated by an individual claw of a class II KC dendrite with multiple small dendritic protrusions (D) and by a spiny class I KC dendrite with one spine‐like protrusion (spine) (E). AL, antennal lobe; CB, central body; GL, glomerulus; KC, Kenyon cell; LA, lamina; LCA, lateral calyx; MB, mushroom body; MCA, medial calyx; ME, medulla; MG, microglomerulus; OL, optic lobe; PN, projection neuron. Scale bars: A: 250 µm; B: 100 µm; B’: 2 µm.

Honeybees emerge into adult life inside a colony guided by social and other multisensory stimuli (mainly olfactory, gustatory, and tactile ones). Following an age‐related polyethism, young adult worker bees first participate in a sequence of different tasks inside the dark hive for about 3 weeks and then leave the nest as foragers to find food and navigate back to the hive (e.g., Robinson 1992; for review, see Johnson 2010). This comprises a switch from duties in the dark hive to an outdoor lifestyle that involves spatial navigation for localizing and memorizing attractive sources for nectar, pollen, or water in a changing olfactory and visual environment (for review, see Menzel and Giurfa 2001). In the brain, the MBs (Figure 1) are multimodal sensory integration centers important for olfactory and visual integration, learning, and the formation of associative memories (e.g., Fahrbach 2006; Giurfa 2007, 2013; Menzel 1999). In honeybees, the MBs comprise approximately 368,000 densely packed intrinsic neurons (Kenyon cells, KCs) that make up more than ∼40% of the total number of neurons in the brain (Rössler and Groh 2012; Strausfeld 2002; Witthöft 1967). Each brain hemisphere comprises one MB divided into paired, cup‐shaped lateral and medial calyces and a shared peduncle terminating in a vertical and a medial output lobe (Figure 1A–C). The MB calyces receive olfactory input from the antennal lobes, visual input from the optic lobes, and, to a minor extent, projections from gustatory neurons (e.g., Gronenberg 2001; Schröter and Menzel 2003). Each MB calyx can be further subdivided into three anatomically distinct compartments: the lip, innervated by olfactory PNs; the collar, innervated by visual PNs; and the basal ring, innervated by PNs of both modalities (e.g., Ehmer and Gronenberg 2002; Galizia and Rössler 2010; Gronenberg 2001; Kirschner et al. 2006) (Figure 1B,C). Within all compartments of the MB calyx, neuronal circuits are organized in distinct modular synaptic complexes called microglomeruli (MG; Figure 1B’,D,E). Each microglomerulus comprises one large presynaptic axonal PN bouton that is predominantly contacted by numerous f‐actin‐rich postsynaptic profiles from KC dendrites (Ganeshina and Menzel 2001; Groh et al. 2004, 2006, 2012; Groh and Rössler 2008, 2011, 2020) (Figure 1B’). A much smaller number of profiles from γ‐aminobutyric acid (GABAergic), octopaminergic, and dopaminergic extrinsic neurons also contact MG (e.g., Blenau et al. 1999; Ganeshina and Menzel 2001; Grünewald 1999; Hammer 1993; Kraft et al. 2023; Zwaka et al. 2018).

In the adult honeybee, KCs can be classified into two distinct classes based on their dendritic specializations in the MB calyx, the size and position of their somata around the cup‐shaped calyx, and the localization of their axonal terminals within the vertical lobe. So‐called class II KCs with unique claw‐like dendritic specializations have been described in the honeybee and in the fruit fly (e.g., Lee et al. 1999; Mobbs 1982; Strausfeld 2002) (Figure 1D). In both species, class II (clawed) KCs arborize in all regions of the MB calyx (Farris 2005; Lee et al. 1999; Leiss, Groh, et al. 2009; Strausfeld 2002; Strausfeld et al. 2003; reviewed in Fahrbach 2006). *Drosophila melanogaster* possesses ∼4000 KCs in total (Aso et al. 2009), all of which are class II KCs. These make up only a small proportion in the honeybee, with 28,000 class II out of a total 368,000 KCs per brain. The remaining 340,000 KCs are class I (spiny) KCs (reviewed in Fahrbach 2006), representing the predominant KC type in the honeybee. In contrast to large dendritic claws along class II KCs, class I KCs have numerous spine‐like protrusions along their dendritic trees in the MB calyx. Earlier investigations have further categorized KCs according to the position and size of their somata (Farris et al. 1999; Mobbs 1982). The outer compact cells described in these studies correspond to class II KCs, and their somata are arranged along the outer margins of the MB calyx cup, flanking the collar and basal ring (Strausfeld 2002; Figure 1B,C). Somata of class I KCs are located inside the MB calyx cup and have been anatomically subdivided into an innermost group (inner compact KCs) with small somata diameters surrounded by a group of KCs with larger somata diameters (inner noncompact KCs). According to Strausfeld (2002), inner compact KCs innervate the basal ring, whereas inner noncompact KCs innervate the collar and lip regions. Axonal projections of both class I and class II KCs form the peduncle. Dendrites of class I KC dendrites are segregated into the lip, collar, and basal ring of the MB calyx (Figure 1C). This separation of sensory input is maintained in the vertical lobes, where their axonal terminals form three distinct divisions (color‐coded in Figure 1C). Those KCs with dendrites in the basal ring terminate in the dorsal division of the vertical lobe, above KCs from the collar. The axons of KCs emerging from the lip terminate in a layer formed in the middle of the vertical lobe. Class I KCs project to both the vertical and medial lobes, whereas class II KCs do not bifurcate and form a large layer in the lowermost third of the vertical lobe, called the gamma (γ) lobe (Strausfeld 2002; for reviews, see Fahrbach 2006; Farris 2005).

KCs of both classes form distinct MG with axonal PN boutons in the MB calyx. Three‐dimensional quantitative imaging and serial electron microscopy studies highlighted MG as important sites of structural neuronal plasticity triggered by sensory input, age‐related processes, and long‐term memory (for a more recent review, see Groh and Rössler 2020). During the interior–exterior transition from nursing to foraging, a volume increase within all compartments of the calyx is caused by massive KC dendritic growth accompanied by PN bouton pruning (e.g., Farris et al. 2001; Groh et al. 2012; Muenz et al. 2015; Scholl et al. 2014). In contrast to the PN bouton pruning following non‐associative sensory exposure, associative olfactory learning and stable long‐term memory were shown to lead to a modality‐specific and volume‐independent increase of PN bouton densities in the MB calyx lip in both the honeybee and in ants (Falibene et al. 2015; Hourcade et al. 2010). This raises the question of how the pre‐ and postsynaptic organization of the circuitry of individual MB calyx MG is adjusted during adult maturation. Three‐dimensional reconstructions of serial electron microscopy brain sections showed that pruning of PN boutons during the nurse‐forager transition is associated with a substantial increase in the number of postsynaptic profiles contacting individual MG by about ∼34% (Groh et al. 2012). This underlines a high level of postsynaptic plasticity in the KC dendritic network. However, a systematic, quantitative analysis of dendritic specializations, their sensory input sites, and their anatomical associations with MG in the two classes of KCs is still lacking.

In the present study, we focused on the following aspects by looking at adult, age‐controlled worker honeybees: How do the two KC classes differ in how they anatomically approach individual presynaptic PN boutons in the MB calyx lip and collar? Are there different dendritic morphologies or specializations within the two KC classes? How do contacts to PN boutons differ, and how does experience and/or adult maturation affect these contacts? To analyze the dendritic specializations of individual KCs and their associations with presynaptic PN boutons, we retrogradely labeled the dendritic network of small subsets of KCs in the MB calyx neuropil via fluorescent tracer injections into specific layers of the VL and combined this with immunolabeling of synaptic proteins to visualize presynaptic PN boutons.

## 2 Materials and Methods

### 2.1 Animals and Age Cohorts

Age‐cohort experiments were conducted in 2022 using *Apis mellifera carnica* workers from a single colony reared outside under natural conditions at the institutional apiary of the University of Würzburg (Germany). Freshly emerged honeybee workers (≤24 h old) were either immediately prepared for retrograde tracer injections (1‐day‐old, see below) or paint‐marked on the dorsal surface of the thorax (around 1500 worker bees) and transferred back into the host colony. Marked honeybees were collected on days 7 and 15 after emergence from inside the hive, while the last age cohort was caught at the hive entrance and identified as ≥28‐day‐old marked foragers by their pollen loads.

### 2.2 Antibody Characterization

To label synapse‐rich PN boutons in the honeybee MBs, a monoclonal antibody against synapsin (SYNORF1, mouse anti‐synapsin; kindly provided by E. Buchner, University of Würzburg, Germany) (Table 1) was used. Synapsin is expressed in presynaptic terminals and is highly conserved across invertebrate species (Hofbauer et al. 2009; Klagges et al. 1996). Anti‐synapsin immunoreactivity allows for visualization and 3D reconstruction of synapsin‐rich neuropils in insect brains (Bressan et al. 2015; Habenstein et al. 2020, 2021, 2023; von Hadeln et al. 2018; Heinze and Reppert 2012; Immonen et al. 2017; Ito et al. 2014). The SYNORF1 antibody was raised in mice against fusion proteins consisting of glutathione‐S‐transferase and the SYN1 protein in *D. melanogaster* (Klagges et al. 1996). The antibody specificity has been characterized in *D. melanogaster* (Klagges et al. 1996) and the honeybee (Pasch et al. 2011). Its affinity was further shown in neuroanatomical studies in various insect species (Grob et al. 2021; Groh and Rössler 2011; Habenstein et al. 2020, 2021, 2023; von Hadeln et al. 2018; Immonen et al. 2017). For the detection of anti‐synapsin, we used the fluorescently labeled secondary antibody CF633 goat anti‐mouse IgG (Cat# 20121, RRID: AB_10582886, Biotium, Fremont, CA, USA).

**TABLE 1:** Primary antibodies.

Antibody	Immunogen	Manufacturer; species; clonality; Cat #; RRID	Dilution
Synapsin	*Drosophila* synapsin glutathione‐S‐transferase fusion protein	E. Buchner, Theodor‐Bovri‐Institute, University of Würzburg, Germany; mouse; monoclonal; Cat #3C11 (SYNORF1); RRID: AB_528479	1:25

### 2.3 Preparation and Retrograde Injections

**FIGURE 2:**
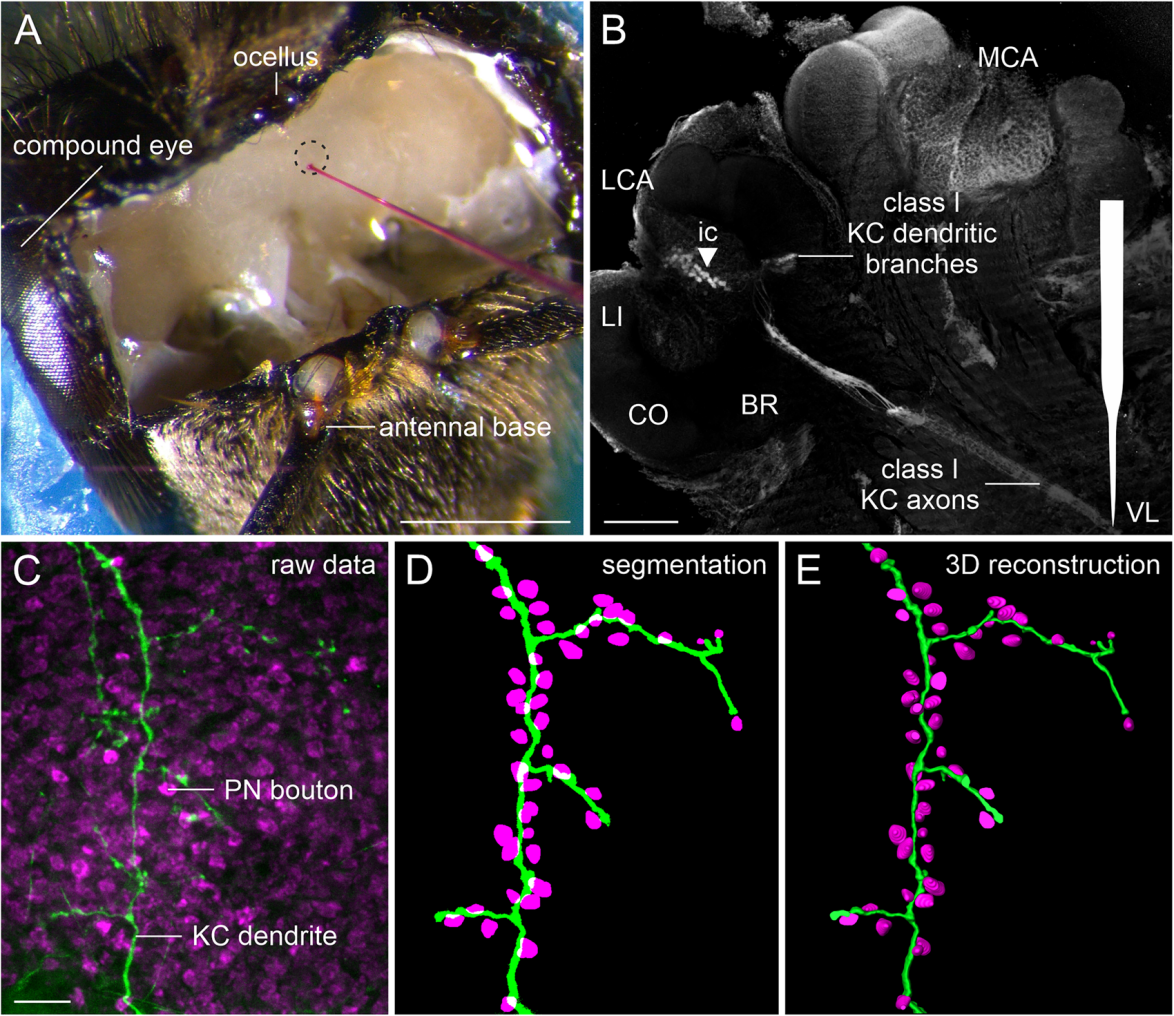
Summary of anatomical methods. (A) Worker honeybee with the head capsule opened between the ocelli, antennal bases, and compound eyes. Fluorescent dye pressure injections into the left VL (indicated by a dashed circle) to retrogradely label small populations of KCs. (B) KC axon bundles run from the VL to the LCA and MCA. Dendritic branches in the BR and a few ic somata (marked with a triangle) are visible. Schematized glass capillary points to the injection site (VL). (C–E) High magnification of a confocal image (*z*‐stack projection) of an individual KC dendrite in the MB calyx lip and its 3D reconstruction. (C) Confocal sections of tissue stained with anti‐synapsin (magenta) and micro‐Ruby enhanced with streptavidin (green). (D) Segmentation of raw data recordings to create 3D reconstructions. (E) Shape and connectivity of KC dendrites were analyzed based on 3D reconstructions. BR, basal ring; CO, collar; ic, inner compact; KC, Kenyon cell; LCA, lateral calyx; LI, lip; MCA, medial calyx; PN, projection neuron; VL, vertical lobe. Scale bars: A: 1 mm; B: 100 µm; C (also applies to D, E): 5 µm.

For retrograde tracer injections, 83 honeybees in total were anesthetized on ice and placed in customized acrylic holders. The head, antennae, and mouthparts were fixed on the holder with dental wax sticks (Surgident, Sigma Dental Systems, Handewitt, Germany). The head capsule was opened by cutting a window between the compound eyes, ocelli, and the antennal base. The exposed brain was rinsed with ice‐cold physiological saline solution (130 mM NaCl, 5 mM KCl, 4 mM MgCl_2_, 5 mM CaCl_2_, 15 mM Hepes, 25 mM glucose, 160 mM sucrose; pH 7.2), and hypopharyngeal glands, fat bodies, and tracheae were removed to access the brain (Figure 2A).

Glass capillaries (1B100F‐4, WPI, Sarasota, FL, USA) were prepared using a DMZ‐Universal‐Electrode‐Puller (Zeitz Instruments GmbH, Martinsried, Germany) and filled with 5% tetramethylrhodamine–biotin dextran (micro‐Ruby; 3000 MW, lysine‐fixable, Cat# D7162, Invitrogen by Thermo Fischer Scientific, Waltham, MA, USA) in phosphate‐buffered saline (PBS; 137 mM NaCl, 2.7 mM KCl, 8 mM Na_2_HPO_4_, 1.4 mM KH_2_PO_4_; pH 7.2). The filled capillaries were affixed to a microinjector (PV820 Pneumatic PicoPump, WPI) using microelectrode holders (MEH6SF, WPI), and their tip was carefully broken. The microinjector was then attached to a manual micromanipulator on a magnetic stand (M3301L and M10, respectively, WPI). The capillary tip was inserted into the VL (dashed circle in Figure 2A), where the micro‐Ruby solution was then pressure‐injected by releasing one short pulse of air at a pressure of 16 psi. The exposed brain was thoroughly rinsed with physiological saline solution, and the piece of cuticle was replaced on the head capsule. Honeybees were kept in a dark, humidity chamber at room temperature for 4 h to let the dye diffuse.

### 2.4 Immunohistochemistry

After the 4h incubation period in the humidity chamber, honeybee heads were mounted in dental wax‐coated dishes (Modelling wax, Dentsply International, York, PA, USA) and brains were dissected in physiological saline solution and subsequently fixed in ice‐cold 4% formaldehyde (FA; methanol‐free, Cat# 28908, Thermo Scientific, Schwerte, Germany) in PBS overnight at 4°C. The brains were washed with PBS solution (5 × 10 min) before being embedded in 5% low‐melting‐point agarose (Agarose II, CAS# 9012‐36‐6, Amresco, Solon, OH, USA) to be sliced in a horizontal plane into 100‐µm sections using a Vibratome (VT 1000S, Leica Biosystems, Nussloch, Germany). All washing steps described were conducted at room temperature on an orbital shaker unless stated otherwise.

Brain sections were permeabilized with 2% and 0.2% Triton‐X 100 solutions (PBST; Cat# A1388, AppliChem GmbH, Darmstadt, Germany; 1 × 10 and 2 × 10 min, respectively). They were then pre‐incubated in 0.2% PBST and 2% normal goat serum (NGS; Cat# 005‐000‐121, RRID: AB_2336990, Jackson, ImmunoResearch Laboratories Inc., West Grove, PA, USA) for 1 h at room temperature. To label synaptic boutons, the sections were subsequently incubated with anti‐synapsin (1:25; Cat# 3C11 [anti SYNORF1], RRID: AB_528479, DSHB; kindly provided by E. Buchner) and Alexa 568 Streptavidin (1:500; Cat# S‐11226, RRID: AB_2315774, Invitrogen by Thermo Fisher Scientific, Waltham, MA, USA) in 0.2% PBST with 2% NGS for 2 days at 4°C. Sections were washed in PBS solution (5 × 10 min) before incubating with the secondary antibody CF633 goat anti‐mouse (1:250; Cat# 20121, RRID: AB_10582886, Biotium, Fremont, CA, USA) and CF488 Phalloidin (1:200; Cat# 00042, Biotium) in PBS with 1% NGS overnight at 4°C. Sections were then washed in PBS solution (5 × 10 min) and transferred to 60% glycerol in PBS (1 × 20 min) before being mounted on glass slides in 80% glycerol in PBS. The slides were sealed with nail polish and stored in the dark at 4°C.

### 2.5 Image Acquisition, Processing, and Data Analysis

Histological preparations were visualized using a laser‐scanning confocal microscope with upright microscope configuration (Leica TCS SP8 MP, Leica Microsystems AG, Wetzlar, Germany) using either a 20x multi‐immersion objective (HC PL APO 20x/0.75 IMM CORR CS2) with 0.75x digital zoom for identification of injection sites and MB overviews or a 63x glycerol immersion objective (HC PL APO 63x/1.30 Glyc CORR CS2) with 3.0x digital zoom for serial scans (*Z*‐stacks) of individual neurons at high spatial resolution. *Z*‐stacks did not have a set depth but rather followed individual neurons as long as possible. We used the 488‐, 552‐, and 638‐nm laser lines (Coherent, Santa Clara, CA, USA) for spectral excitation. All images were taken at a resolution of 1024 × 1024 pixels. *Z*‐stacks were taken using a 0.33‐µm step size. To compensate for the loss of fluorescence intensity throughout the entirety of each *Z*‐stack, the detector gain was adjusted.

Initial image processing was conducted using the Fiji ImageJ plugin (version 1.54f; National Institutes of Health [NIH], RRID: SCR_003070). The channels in triple‐stained brain sections were split to easily identify individual elements such as KC neurites and PN boutons, and *Z*‐stack projections were conducted (Figure 2B,C). Brightness and contrast were adjusted when needed.

After initial assessment, dendritic arbors of individual KCs were further processed using the 3D software Amira (version 2019.1; RRID: SCR_007353, Thermo Fisher Scientific). PN boutons and KC dendrites were separately reconstructed using a semiautomated threshold in the *Image Segmentation Editor*. In the *Segmentation Editor*, label fields were created based on the raw stacks of the individual PN bouton and KC dendrite channels. Using the *Threshold* tool, voxels were automatically classified into “foreground” (i.e., the structures of interest) and “background” depending on their intensities (0–255). The range of desired intensities could be manually edited, while, in cases where voxels not belonging to the structure of interest were assigned to the “foreground,” the *Brush* tool was manually used to assign them to the “background.” The processed segmentation was then extracted and visualized as an orthogonal slice (Figure 2D). The segmented surface module was visualized in 3D using the *GenerateSurface* and *SurfaceView* modules (Figure 2E). To calculate the frequency of putative spine–bouton contacts in class I KCs, the length of KC dendrites was measured using the 3D length tool. To measure using the 3D length tool, we zoomed in on small regions of the dendrite and drew small lines corresponding to the 3D surface of the dendrite and its dendritic specializations. Once the whole dendrite was measured, the values were exported into a spreadsheet and extracted into a CSV file, where they were then added together to acquire the total length of the dendrite. The number of anti‐synapsin‐labeled PN boutons putatively contacting the KC dendrite was also counted using the *Landmarks* tool. A threshold distance for putative synaptic‐spine contacts was defined (as in previous studies) to be at a distance of ∼0.3 µm (Sätzler et al. 2002; Zwaka et al. 2016). The frequency of putative spine–bouton contacts per 10 µm dendrite length was then determined along the total length of the respective dendrites. For class II KCs, the enwrapping percentage, determined as the percentage of the PN bouton area covered by individual claws, was analyzed as follows: synapsin labeling was used to estimate surface areas (µm^2^) of individual PN boutons. The claw surface was then removed to calculate the non‐enwrapped surface area of a PN bouton. The non‐enwrapped percentage was calculated by dividing the non‐enwrapped surface area by the whole surface area of a PN bouton multiplied by 100. The enwrapping percentage was calculated by subtracting the result from 100.

## 3 Results

### 3.1 Injection Sites in the Vertical Lobe and KC Somata Localizations

**FIGURE 3:**
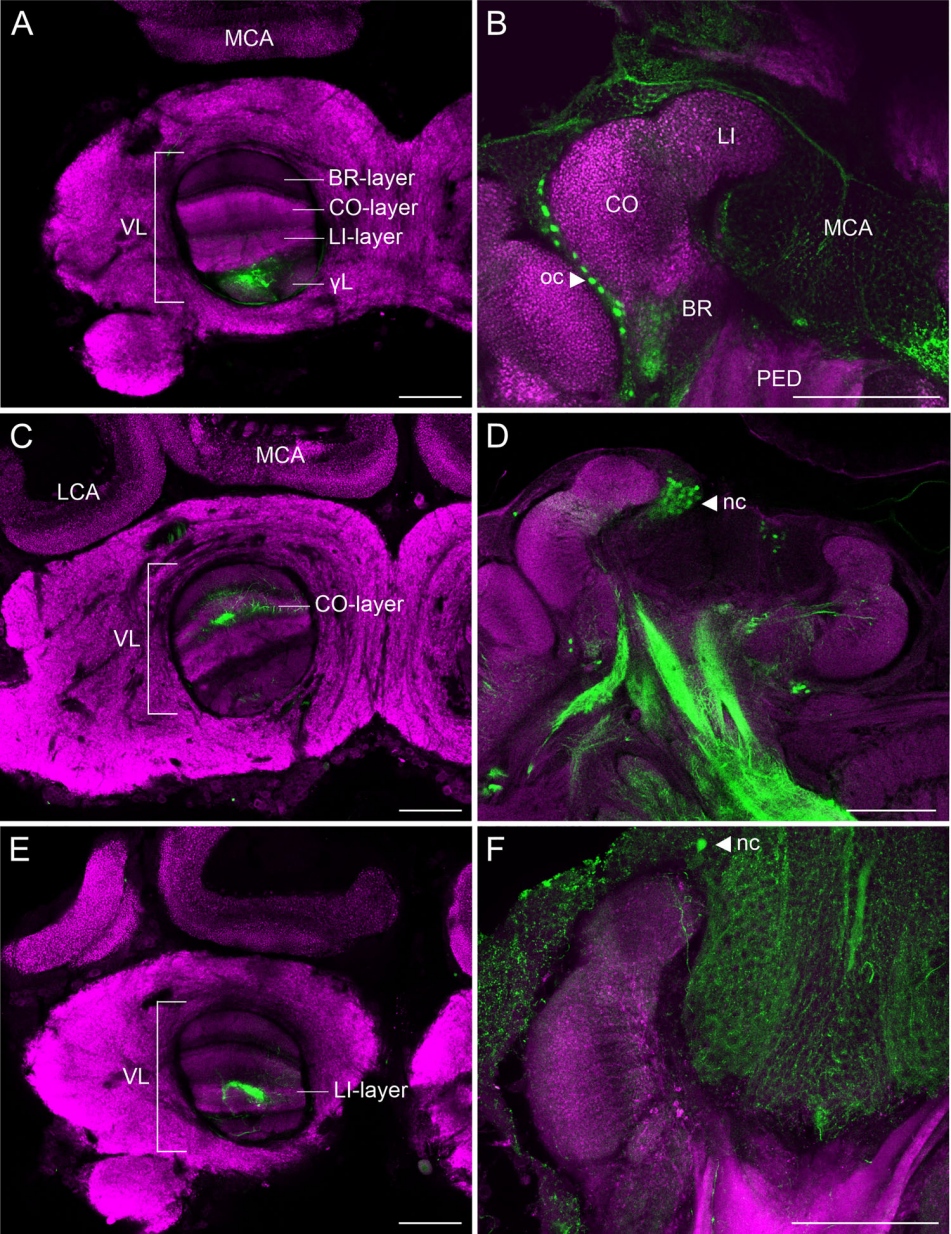
Injection sites in the VL and correspondence with localization of stained KC somata surrounding the CA neuropil. Brains stained with anti‐synapsin (magenta) and micro‐Ruby enhanced with streptavidin (green). (A, C, E) Examples of injection sites at the γL (A), CO (C), and LI (E) layers of the VL are shown. (B, D, F) Triangles indicate the respective position of outer compact (B) and noncompact (D, F) KC somata. BR, basal ring BR‐layer of the VL corresponding to the basal ring; CO, collar; CO‐layer of the VL corresponding to the collar; γL, gamma lobe of the VL; LCA, lateral calyx; LI, lip; LI‐layer of the VL corresponding to the lip; MCA, medial calyx; nc, noncompact; oc, outer compact; PED, peduncle; VL, vertical lobe. Scale bars: 100 µm.

Micro‐Ruby injected into axons of individual or small populations of KCs retrogradely labeled their dendritic arbors and somata in the MB calyx. For proper identification and classification of KC classes, the injection sites in distinct divisions of the vertical lobe, together with the location of the respective KC somata relative to the MB calyx cup, were used as parameters for anatomical identification (Figure 3). The staining was successful in 44 bees in total and usually comprised small or larger groups of KCs (ranging between 1 and a few hundred somata per 100 µm section), which was verified by the number of KC somata labeled. Single dendritic branches of individual KCs from labeled groups were then selected for analysis based on their visual accessibility. In some cases, the staining, though successful, comprised bundles of KCs that were too densely packed for further analysis. Out of 25 confocal stacks from successfully stained class I and II KCs, 11 were suitable for further 3D analysis.

Following tracer injection into the γ‐lobe, outer compact cells corresponding to class II KCs were stained outside the MB calyx cup (Figure 3A,B), while tracer injections into the three upper vertical lobe divisions labeled class I KCs with somata inside the MB calyx cup (Figure 3C–F). Our analyses were focused on class I and II KCs associated with the MB calyx collar and lip. In the basal ring, KC processes were extremely densely packed, and individual dendrites could not be extracted at the optical resolution available. Therefore, the distinction of lip and collar KCs from those of the basal ring was important. For this, injection sites in the corresponding collar and lip divisions of the vertical lobe (Figure 3C and 3E, respectively) were evaluated, and noncompact KC somata belonging to class I KCs innervating the collar and lip were identified (Figure 3D and 3F, respectively). The distinction between clawed KCs innervating the lip and collar from those innervating the basal ring was carried out by tracking the dendritic arborizations of selected KCs into the lip or collar regions of the MB calyx. The pre‐evaluations of staining results were essential initial steps prior to further analyses.

### 3.2 Main Dendritic Morphologies in Class I and II KCs

To analyze dendritic specializations and characterize the anatomical connectivity between KC dendrites and PN boutons in the MB calyx, raw confocal images were first visually evaluated for identification of dendritic specializations and then further processed. Differences in overall morphology and dendritic specializations across and within the two KC classes were already evident in raw data images.

**FIGURE 4:**
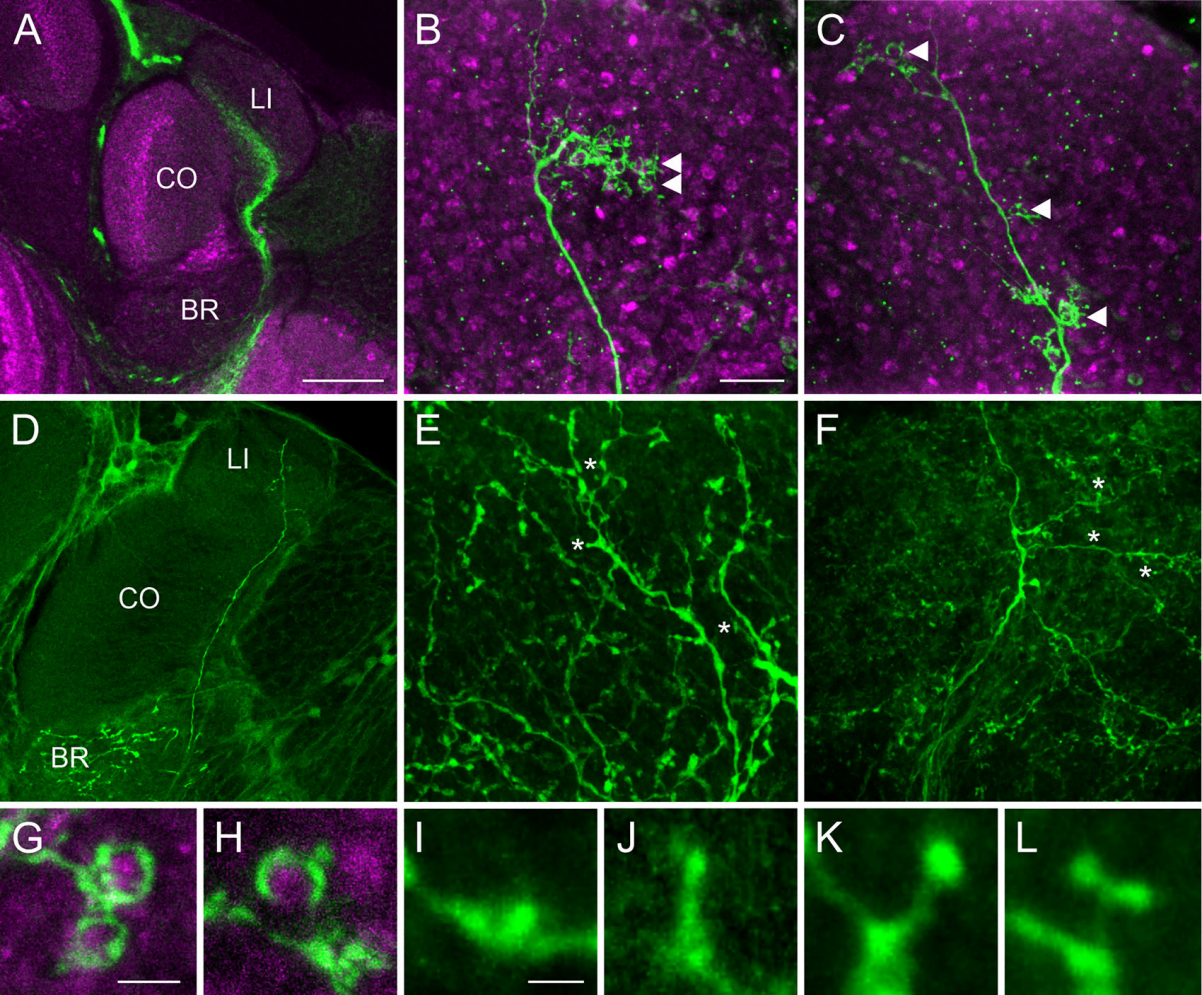
*Z*‐projections of confocal images of the two different classes of KCs and their dendritic specializations. Sections are labeled with anti‐synapsin (magenta) and micro‐Ruby enhanced with streptavidin (green). (A–C) Clawed KC dendrites run through the MB calyx lip (A, single optical section) and comprise either a single cluster of claws (B, stack of 62 optical sections) or several small dendritic clusters (C, stack of 75 optical sections). Triangles indicate single claws. (D–F) Branching patterns of class I KC dendrites from different honeybees. D: single optical section; E: stack of 69 optical sections; F: stack of 91 optical sections. Asterisks indicate dendritic specializations of different classes coexisting in individual dendrites. (G, H) Close‐ups of individual claws indicate that claws enwrap different surface areas of PN boutons. G: stack of 10 optical sections; H: stack of 15 optical sections. (I–L) Four categories of class I KC dendritic specializations: (I) stubby, stack of nine optical sections; (J) thin, stack of eight optical sections; (K) mushroom, stack of eight optical sections; and (L) branched, stack of 11 optical sections. Classification after Leiss, Koper, et al. (2009). BR, basal ring; CO, collar; LI, lip. Scale bars: A (also applies to D): 50 µm; B (also applies to C, E, F): 10 µm; G (also applies to H): 2.5 µm; I (also applies to J–L): 1 µm.

Confocal image analyses revealed that dendrites of individual class II KCs innervate either the lip or the collar and never bifurcate into both compartments (Figure 4A). Innervation of the basal ring by collateral branches of individual class II KCs, however, could not be ruled out (Figure 4A). Class II KC dendrites possessed clusters of dendritic branches with claw‐like specializations (from here on referred to as “claws”) enwrapping individual PN boutons. Such clusters of dendritic branches either occurred as single clusters comprising many claws in close vicinity (class IIa; Figure 4B) or as multiple clusters with sparse claws distributed along the main dendrite (class IIb; Figure 4C). The somata of class IIa and IIb types were not positioned in distinct manners and were even observed to be adjacent to one another. Single PN boutons appeared to be enwrapped to different degrees by individual claws (Figure 4G,H). There was a clear overlap of micro‐Ruby and phalloidin labeling around PN boutons (Figure 1B’). Most importantly, individual PN boutons were always putatively contacted by only one claw of an individual dendrite.

Staining of class I spiny KCs confirmed strict segregation of their dendrites into the lip and collar of the MB calyx, depending on the injection sites in respective vertical lobe divisions. Visual analysis of 15 individual class I KC dendrites showed that they possessed multiple distributed branches that sometimes covered a substantial area within an MB calyx compartment (Figure 4D). Class I KC dendrites were often characterized by relatively thick, varicose specializations (stubby; Figure 4I), which we never found along class II KC dendrites. In addition, spine‐like protrusions could be classified into thin, mushroom, and branched following the nomenclature by Leiss, Koper, et al. (2009) for dendritic spines of lobula plate tangential cells of the *Drosophila* visual system (Figure 4J–L). These different dendritic specializations coexisted in individual class I KC dendrites (asterisks in Figure 4E,F; and see below) and were identified as such by scrolling along the *Z*‐axis and later via 3D reconstructions.

### 3.3 Exemplary 3D Reconstructions of Dendritic Specializations in Class II KCs

For high‐resolution 3D reconstructions, individual class II KCs with claws clearly belonging to a single dendrite were selected from confocal images from two successfully stained adult bees of unknown age. The main selection criterion was a long stretch of an individual dendrite that could be clearly separated from small groups of other labeled KC dendrites. The confocal data were further processed for 3D reconstruction, allowing a more detailed, quantitative analysis of the putative anatomical associations between PN boutons and postsynaptic KC claws.

**FIGURE 5:**
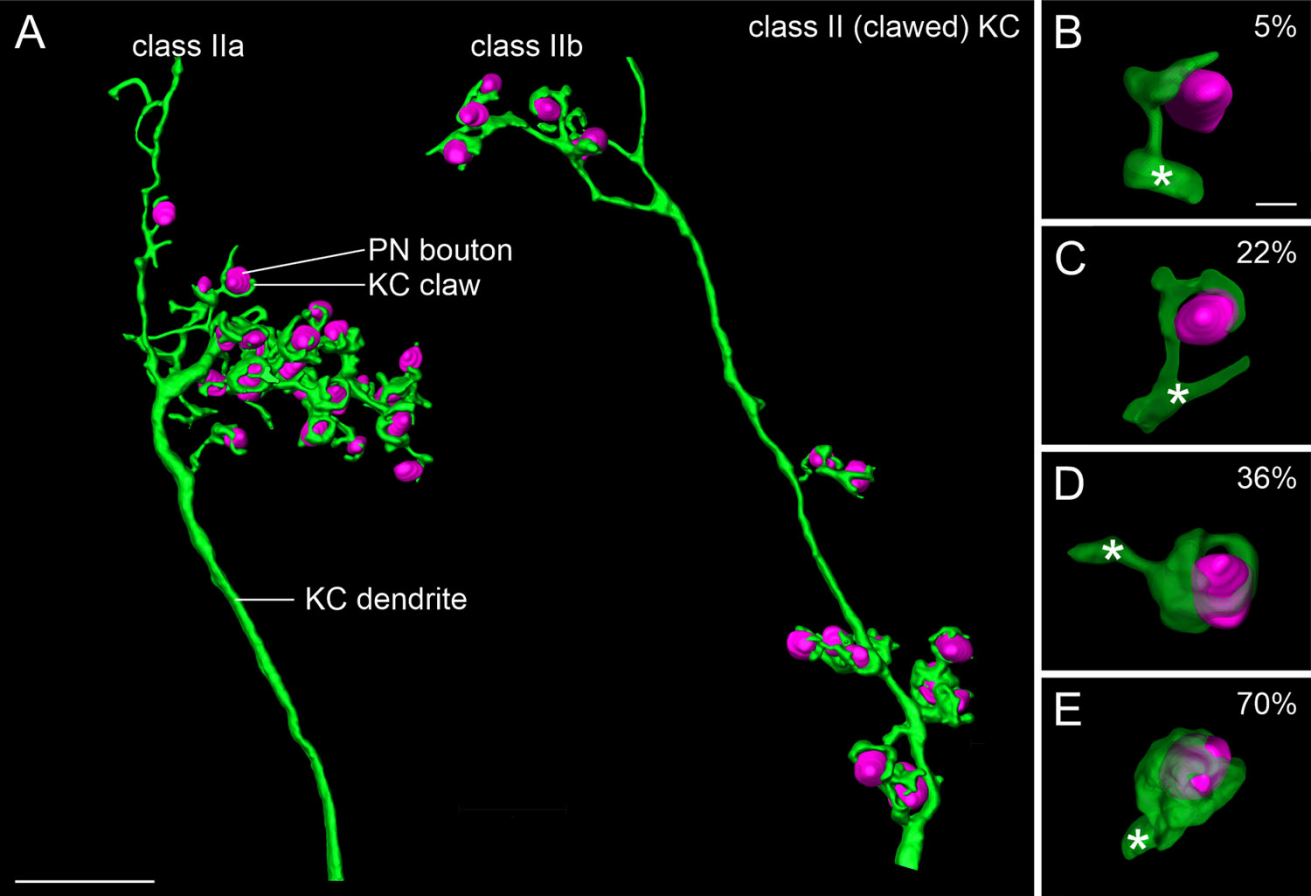
Examples of 3D reconstructions of class II KC dendrites and their putative associations with PN boutons. (**A)** Examples of class II KC dendrites exhibiting either a big local cluster of claws (type IIa; left) or claws distributed on multiple smaller dendritic clusters (type IIb; right). (**B–E)** Different percentages of PN bouton surfaces enwrapped by dendritic claws. Asterisks mark the KC dendrite. KC, Kenyon cell; PN, projection neuron. Scale bars: A: 10 µm; B (also applies to C–E): 1 µm.

The 3D reconstructions revealed that the main dendrites of class II KCs were rather smooth or uniform before arborizing into clusters of claws (Figure 5A). Further qualitative analysis highlighted differences in individual arborizations. Although some dendrites exhibited clusters with numerous claws within a relatively small volume of dendritic branches (Figure 5A, left; class IIa), others possessed smaller clusters of dendritic branches distributed along the main dendrite with much fewer claws in each cluster (Figure 5A, right; class IIb). The morphology of claws did not differ between the two types of dendritic arborization patterns. In both cases, single PN boutons were putatively contacted by only one claw emerging from an individual dendrite.

Three‐dimensional reconstructions confirmed that individual claws showed a wide range of morphological variations regarding putative anatomical contacts with PN boutons (Figure 5B–E). In some cases, claws contacted PN boutons unilaterally (Figure 5B), whereas in other cases, they formed an almost toroid‐like structure around the entire PN bouton (Figure 5D,E). Claws from individual dendrites widely differed regarding the surface areas covered on PN boutons. The enwrapping degree was further analyzed as the percentage of PN bouton surface area covered by a claw. This ranged between ∼5% and 70% (*N* = 41 claws from two class II KCs) with an average percentage of 27%. Across all reconstructed KC claw and PN bouton pairs, the analyses revealed that a PN bouton was never entirely enwrapped by an individual KC claw.

### 3.4 Exemplary 3D Reconstructions of Dendritic Specializations in Class I KCs

Confocal image stacks from an overall number of five class I KCs in the MB calyx collar and four in the lip were reconstructed in 3D. Like in class II KCs, the main selection criterion was a long stretch of an individual dendrite that could be clearly separated from small groups of labeled KC dendrites. We selected examples from different age cohorts to screen for potential age‐related differences in spine–bouton associations (Table 2).

**TABLE 2:** Number of putative spine–bouton contacts in class I KCs in different age cohorts of worker bees. The number of putative spine–bouton contacts per 10 µm dendrite length was determined by dividing the number of spine–bouton contacts by the length of the dendrite (spines/µm) multiplied by 10.

Age (days after emergence)	MB calyx compartment	Dendrite length (µm)	Total number of putative spine–bouton contacts	Putative spine–bouton contacts (10 µm)
1	Collar	231.78	58	2.5
	Lip	64.30	25	3.9
		262.62	88	3.4
8	Collar	120.63	36	2.9
	Lip	179.78	45	2.5
15	Collar	260.80	49	1.9
		150.18	26	1.7
≥28	Collar	186.34	40	2.2
	Lip	220.19	54	2.5

**FIGURE 6:**
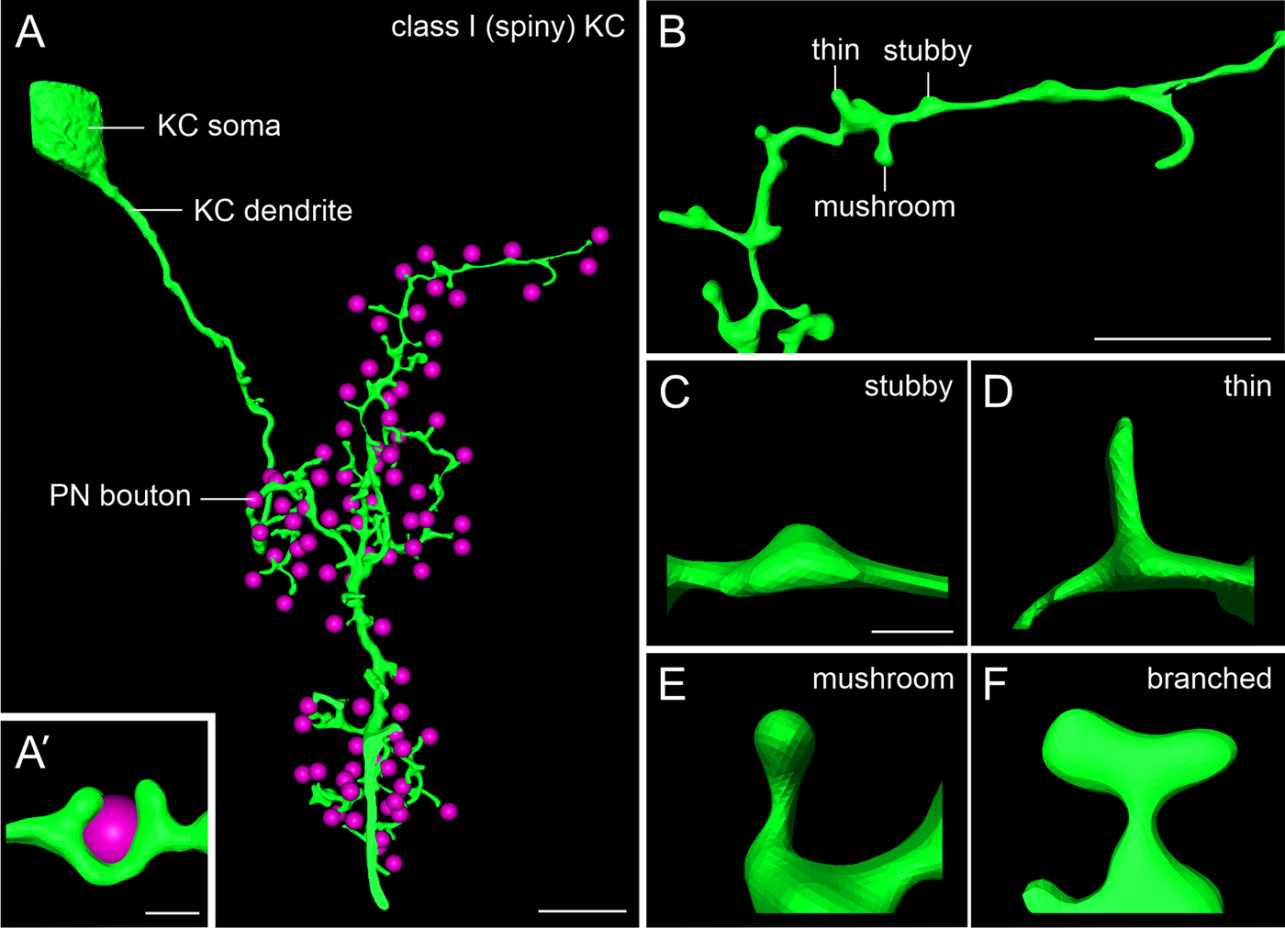
Exemplary 3D reconstruction of class I KC dendrites and their putative associations with PN boutons. (A) A single dendritic specialization from one KC dendrite putatively extends to a single presynaptic PN bouton. (A’) A singular (rare) case of two neighboring spines on the same dendrite putatively contacting one PN bouton. (B) Different categories of spines can be found along one dendrite (e.g., thin, mushroom, and stubby). (C–F) Dendritic specializations can be subdivided into four morphological categories: (C) stubby, (D) thin, (E) mushroom, and (F) branched. KC, Kenyon cell; PN, projection neuron. Scale bars: A: 10 µm; A’: 1 µm; B: 5 µm; C (also applies to D–F): 1 µm.

Class I KCs varied in thickness (see examples in Figure 4E,F). This applies to dendrites within the same MB calyx compartment and to bees of different ages. Some KC dendrites were thick with only a few branches, while others were thin and had densely branched arborizations (see example in Figure 6A). The majority of KC spines extended almost perpendicularly to the longitudinal axis of the main dendritic branch (e.g., Figures 4E,F and 6A,B). In almost all cases we examined, only one spine from a bypassing class I KC dendrite extended to an individual PN bouton. Only in one case (one out of 421 PN boutons analyzed), we found a PN bouton that was putatively contacted by two neighboring dendritic spines of the same individual KC dendrite (Figure 6A’). The typical one‐to‐one relationship between PN boutons and class I KC spines was independent of age (420 of 421 PN boutons analyzed across all age cohorts).

We subdivided dendritic spines of class I KCs into four distinct morphological categories. These were found across all analyzed age cohorts and MB compartments. In many cases, the four morphologies were found along the same KC dendrites (Figure 6B). High‐magnification 3D reconstructions highlight the four morphological spine categories: stubby, thin, mushroom, and branched (Figure 6C–F; nomenclature after Leiss, Koper, et al. 2009). The four morphological categories did not occur at the same frequency; branched spines were rarely observed (four branched spines in total across all age cohorts), while the other three morphological categories were more common and varied depending on the complexity of the KC dendrite.

To examine potential age‐related changes in the PN bouton to spine connectivity across cohorts, we calculated the frequencies of putative spine contacts (number of spines associated with PN boutons per 10 µm of dendrite length) according to our definition for putative spine–bouton contacts (see methods section). The time‐consuming reconstructions were done for an exemplary subset of 3D‐reconstructed individual class I KC dendrites and their associated PN boutons in the lip and collar (*N* = 9) across the four age cohorts (Table 1). In these examples, the number of putative spine–bouton contacts per 10 µm dendrite length ranged between 1.73 and 3.89 but did not show any obvious trend for age‐related differences in spine–bouton frequencies.

## 4 Discussion

We analyzed anatomical relationships between PN boutons and KC dendritic specializations at the level of individual MG across and within the two KC classes in the MB calyx of adult worker honeybees. Using confocal microscopy analyses of retrograde tracings combined with anti‐synapsin immunolabeling and 3D reconstructions, we characterized dendritic branching patterns and specializations in class I and II KCs. In class II KCs, we found two morphological subtypes with either locally clustered or more distributed dendritic branching patterns, together with a strong variation in the enwrapping range of PN boutons by dendritic claws. Importantly, dendrites of all class II (clawed) KCs we investigated were clearly segregated into either the lip or collar. We did not find any class II KC that extended arbors through both the lip and collar. We classified four types of spine shapes in class I KCs and showed that these types were found throughout all age cohorts investigated. Our results further show that individual PN boutons are predominantly contacted by only one spine or claw from a bypassing dendrite, and this one‐to‐one relationship was found across all age cohorts. Finally, results from exemplary class I KC dendrite and PN bouton reconstructions indicate that the frequency of spine–bouton associations remains rather constant (∼2–3 per 10 µm) across all age cohorts investigated.

### 4.1 Correspondence of Injection Sites and KC Somata Locations

Anatomical correlations of the injection sites in the vertical lobe and locations of labeled somata were essential for proper classification of KC identities. The results confirmed the localization of outer compact, noncompact, and inner compact KC somata with respect to their axonal terminals in the different vertical lobe divisions. This also allowed us to distinguish between class I and II KCs dendrites arborizing in the MB calyx lip and collar. The results are consistent with the somata localization and vertical lobe divisions revealed earlier based on Golgi staining techniques (Farris et al. 1999; Strausfeld 2002).

### 4.2 General Characterization and Dendritic Specializations of Class II KCs

Clawed KCs, albeit the minority in the MB calyx of the honeybee, represent the only or predominant type of intrinsic neurons in the MB of other insect species (*Acheta domesticus*: Schürmann 1970; *D. melanogaster*: Lee et al. 1999; *Periplaneta americana*: Strausfeld and Li 1999; *Spodoptera littoralis*: Sjöholm et al. 2005). In the honeybee, clawed KCs extend dendrites into all compartments of the MB calyx, and their axons terminate in the γ‐lobe division of the vertical lobe, irrespective of the localization of their dendritic input in the different calycal compartments (Strausfeld 2002). With our methods, we wanted to clarify whether individual class II KC dendrites may arborize in both the lip and collar, potentially receiving convergent input from visual and olfactory modalities. Our results revealed that none of the class II KC dendrites (from a total of 35 class II KCs that were visually analyzed) had dendritic arborizations in both the lip and collar. In all cases, dendritic innervation was clearly restricted to either the lip or the collar. This indicates that input from olfactory PNs to the lip and from visual PNs to the collar does not converge at the level of class II KC dendrites. However, this may be different in the basal ring, where axonal projections from olfactory and visual PNs terminate in adjacent layers that are in close vicinity (e.g., Mobbs 1982; reviewed in Gronenberg 2001). Our tracing technique and the limitations of optical resolution during analyses did not allow differentiation of the fine morphology of individual KC dendrites within the basal ring regions (see Figure 3B). Although axonal terminals from PNs of both modalities remain segregated in distinct layers within the basal ring (Ehmer and Gronenberg 2002; Kirschner et al. 2006), their close spatial vicinity may represent a potential site for anatomical convergence of both modalities on KC dendrites. Future experiments using techniques like electron microscopy or super‐resolution microscopy are necessary to investigate a potential anatomical convergence on KC dendrites in the basal ring at the required level of detail.

We found two distinct types of class II KCs regarding the clustering of dendritic branches: class IIa, with a small number of large, localized claw clusters, and class IIb (also typical for the fruit fly; Leiss, Groh, et al. 2009), with several small clusters of dendritic branches that are spatially distributed and contain fewer claws (Figure 5A). The different morphologies are highly suggestive of functional differences in sensory processing. In the honeybee, visual input from the medulla and lobula is further segregated into layers within the collar and basal ring (Ehmer and Gronenberg 2002), and olfactory input from PNs of the medial and lateral antennal lobe tracts is also segregated into layers within the lip and basal ring (Kirschner et al. 2006). Therefore, single large, localized claw clusters of the IIa type probably receive information from only one layer of segregated PN boutons within a calycal compartment, while smaller, distributed clusters of the IIb type are more likely to receive convergent input from more than one layer of segregated PN boutons.

Analysis of individual class II KC claws at higher optical resolution revealed a wide range in the PN bouton surface covered by individual claws (between ∼5% and 70%). These findings indicate that synaptic efficacy may be regulated at this anatomical level. A large PN bouton surface area enwrapped by a single claw may contact more presynaptic active zones compared to a claw with a smaller enwrapping degree, resulting in a higher likelihood of triggering a postsynaptic response in a PN bouton. In addition, we never observed more than one claw from the same dendrite of a class II KC contacting the same presynaptic PN bouton. In *D. melanogaster*, where clawed KCs are the only type of KCs, individual KCs typically comprise a mean of five to six claws (Butcher et al. 2012; Zheng et al. 2022), with each claw contacting a different single PN bouton (Leiss, Groh, et al. 2009). Furthermore, individual claws never enwrap the complete surface area of a PN bouton, indicating that more than one claw from several KC dendrites innervates a PN bouton (Leiss, Groh, et al. 2009). Although the number of claws along a class II KC dendrite in the honeybee is higher and shows a wider range of morphological variation compared to *Drosophila*, the innervation of one PN bouton per claw is also supported by our findings. Ultrastructural investigations in *Drosophila* showed that, on average, 11–14 claws (from 11–14 KCs, respectively) contact a single PN bouton (Baltruschat et al. 2021; Butcher et al. 2012), and each claw contacts eight to 25 presynaptic sites (active zones) per PN bouton (Baltruschat et al. 2021). Groh et al. (2012) showed that individual PN boutons in the honeybee MB lip may house up to 70 active zones compared to around 40 in the collar in a 1‐day‐old worker bee. Assuming that claws in the honeybee contact the same number of active zones per PN bouton as in the fly and that only one claw from a bypassing class II KC dendrite contributes to an MG, an olfactory PN bouton could be contacted by up to nine and a visual PN bouton by up to five claws from different clawed KCs. A claw that enwraps 70% of a bouton surface area might therefore have larger numbers of thin postsynaptic protrusions and not allow for many more postsynaptic partners. On the other hand, claws covering only 5% of the bouton surface would contact a smaller number of active zones. The wide range of the enwrapping degrees by different claws indicates that this anatomical feature might be dynamically adjusted according to the history of sensory input. This is further supported by the overlap of claws with phalloidin labeling, suggesting that individual claws are highly enriched in f‐actin and are therefore structurally plastic. Future connectome studies using super‐resolution light microscopy (e.g., Kraft et al. 2023) or serial electron microscopy are necessary to clarify these aspects.

### 4.3 Morphological Categories of Dendritic Specializations in Class I KCs

We found four distinct morphological spine categories (stubby, thin, mushroom, and branched) in class I KCs that coexist along individual dendrites and throughout all investigated age cohorts. Dendritic spines are important sites of structural plasticity associated with learning and long‐term memory across vertebrates and invertebrates (reviewed in Sorra and Harris 2000). They are conventionally subdivided into the same four morphological categories we have found in the honeybee spiny KCs and are suggested to represent different levels of maturation and morphofunctional states (*Rattus norvegicus*: Harris et al. 1992; *D. melanogaster*: Leiss, Koper, et al. 2009).

Dendritic spines have most notably been described in rodent hippocampal neurons, particularly their plasticity during behavioral maturation (Harris et al. 1992; Nägerl et al. 2004). This has not been investigated in similar depth in insects. In the *Drosophila* MB, only one type of KCs has been described (clawed), but spiny dendrites have been found in other parts of the fly's nervous system, for example, in neurons of the lateral horn (Yasuyama et al. 2002) and in lobula plate tangential cells (Leiss, Koper, et al. 2009). Combining genetic markers and immunohistochemistry, Leiss, Koper, et al. (2009) found four morphological spine categories along tangential cell dendrites, similar to those previously described in vertebrates. Spiny KCs have also been observed in the MBs of other insects (e.g., *Schistocerca americana*: Laurent and Naraghi 1994; *Spodoptera littoralis*: Sjöholm et al. 2005; for review, see Schürmann 2016). In *S. littoralis*, a moth species, spines are described as “short” and “stubby” (Sjöholm et al. 2005), while in the locust, spines are described as ranging “from elongated and thin to short and stubby” (Laurent and Naraghi 1994). Schürmann (2016) concluded that “abundant tertiary and quaternary branches decorated with dendritic spines” can be found in the brains of crickets and cockroaches. In previous work, they were differentiated as “spines” and “blebs” (Schürmann 1974).

The two classes of KCs in the honeybee MB, compared to only one class in *Drosophila*, raise the interesting question of why two types coexist in the same brain compartments of one species but not in the other. Farris and Schulmeister (2011) argue that the expansion and complexity of hymenopteran MBs coincided with the rise of parasitoidism in wasps and predated sociality and central‐place foraging within the taxon, as there was already a need for associative learning, place, and long‐term memory. The existence and even predominance of spiny KCs in the honeybee may be a consequence of the strong demand for long‐term associative memory, both in the olfactory and visual pathways of central‐place foraging social insects, possibly further evolving from pre‐adaptations of MB neuroarchitecture observed in parasitoid wasps.

In the honeybee, systematic characterization of dendritic spines has been attempted in earlier Golgi studies, for example, by Brandon and Coss (1982) and Dobrin et al. (2011). Brandon and Coss (1982) compared “flyer” and “non‐flyer” groups of honeybees of the same age and measured various morphological parameters. They found a shortening of the spine stem in flyers compared to non‐flyers of the same age and interpreted the enlargement of the spine heads as a consequence of experience. This may be supported by vertebrate literature suggesting that mushroom spines are more mature (Harris et al. 1992). Dobrin et al. (2011) classified spines into “mushroom, filopodia‐like, branched, and tooth” and analyzed spine densities. Both studies used Golgi impregnations, in which spines look different due to strong dehydration during histochemical treatments compared to mild histochemical treatments and confocal microscopy imaging in Leiss, Koper, et al. (2009) and our present study. Interestingly, the spine lengths Leiss, Koper, et al. (2009) reported are also comparable to those in our study. Our results furthermore confirm the morphological types they found, which is why we adapted the nomenclature from their study. The four spine categories are present throughout all investigated age groups and, in many cases, co‐exist on the same dendritic branch (Figure 6B), very much like shown in vertebrates and *Drosophila* (Harris et al. 1992; Leiss, Koper, et al. 2009). This suggests that spine morphology might be rather dynamic and may transform between the different morphological categories. A study on mouse pyramidal cells proposes the lack of strict morphological categories in a way that they have been classically described and offers a “continuum of spine morphologies” as an alternative (Ofer et al. 2021). Another possibility is based on filopodial dynamics. Özel et al. (2015) tracked changes in growing nerve endings in the *Drosophila* brain. They subdivided filopodial growth into layer separation, transient, and stable filopodia, with the transitions between these stages being dynamic. Similarly, dendritic spines in the honeybee may transition while “searching” for appropriate presynaptic partners on PN boutons. This may be triggered by, for example, the increase in MG numbers following stable long‐term memory formation (Hourcade et al. 2010). It would be highly interesting to find out whether these features differ between clawed and spiny KCs in the honeybee MB. Ideally, this should be observed using time‐lapse recordings in a developing system, but a more fine‐grained analysis of a time series of static images may be another option to investigate these aspects in the future.

### 4.4 Frequency of Putative Spine–Bouton Contacts in Class I KCs and Age‐Related Changes in Microglomerular Circuits

We chose class I KCs for exemplary analyses of putative spine–bouton associations and how these may change with age and experience. As the 3D reconstruction of dendritic specializations and their associations with PN boutons in the calyx is very time‐consuming, we did an exemplary analysis of a selected group of class I KC dendrites in both the lip and collar (see Section 2 and Table 2). Interestingly, the frequency of putative spine–bouton contacts (defined as close appositions of less than 0.3 µm) was rather constant with two to three spines per 10 µm across all investigated age cohorts. Groh et al. (2012) showed that the number of postsynaptic partners contacting individual MG increases by 34% when comparing 1‐day‐old bees and experienced foragers. Since our study shows that a bypassing individual dendrite from class I KCs extends only one spine to a PN bouton, the increase in postsynaptic profiles should be from additional dendrites that approach a bouton. This is in line with the observation by Farris et al. (1999) who showed an age‐ and experience‐related increase in major dendritic branches with increasing age. Farris et al. (2001) found a constant spine density between nurses and foragers, but at an overall higher frequency of about nine to 10 spines per 10 µm. This discrepancy may be for two reasons. First, Farris et al. (2001) analyzed Golgi impregnated tissue that has a much higher shrinkage factor than the method we used without dehydration. Second, in contrast to the total number of spines per length estimated by Farris et al. (2001), we counted putative spine–bouton contacts per dendrite length. We believe that spine–bouton contacts are more instructive in estimating differences in PN–KC innervation patterns related to MG structural plasticity.

Previous studies showed a reduction in the overall number of MG (pruning) in experienced foragers compared to young bees, and this reduction gives more room for new ingrowing dendritic branches (Muenz et al. 2015). Dobrin et al. (2011) analyzed spine density in 7‐day‐old bees and foragers by relative location (proximal, medial, distal dendrites) in the MB collar. Although the spine density differed between the relative locations, it remained constant within the individual locations, regardless of age. Altogether, this supports the scenario that novel dendritic branches grow out in between MG and extend new spines to existing PN boutons. The result of this is an increase in the PN to KC ratio, which would also be in line with the function of the MBs as associative memory devices, particularly in the context of long‐term memory (Hourcade et al. 2010).

In vertebrate literature, Golgi studies revealed spine densities of 10–14 spines per 10 µm dendritic length, depending on the brain area and species (Benavides‐Piccione et al. 2002), and more recent EM studies revealed an even higher spine density (up to ∼50 spines per 10 µm) (Kasthuri et al. 2015). These values can change with age, but the changes are highly variable depending on spine category and brain region (reviewed in Dickstein et al. 2013). Dickstein et al. (2013) looked at several vertebrate species and argue that spine density is reduced with age, but the reduction is much more pronounced in thin spines, while stubby or mushroom spines remain relatively constant. Such age‐related structural changes could influence neuronal plasticity and, therefore, memory and learning.

Regardless of age cohort, we show that in almost all cases, a bypassing individual class I dendrite extends only one spine to a PN bouton. Based on this result and the fact that previous studies in the honeybee have speculated multiple postsynaptic partners for individual PN boutons (Groh et al. 2012), it is very likely that a single PN bouton is contacted by spines from potentially many KC dendrites. EM data in the MB calyx in a 1‐day‐old bee showed that the average number of active zones is about 70 for olfactory and about 40 for visual PN boutons, and they form mainly dyads with postsynaptic structures such as KCs (Groh et al. 2012). Assuming all postsynaptic partners would exclusively be class I KCs, we can roughly calculate that one PN bouton may diverge to up to 140 KCs in the lip and to about 80 KCs in the collar. In this calculation, however, claws with small spine‐like protrusions and extrinsic neurons that may also contribute to the postsynaptic site of MG are not included, which may, as a result, inflate our estimates for the contribution of class I KCs.

The pronounced age polyethism and the drastic changes along a honeybee's life history provide a unique system in which sensory input and age can be used as variables to tackle open questions regarding the plasticity of high‐order sensory information processing and long‐term memory circuits. Future use of super‐resolution light microscopy, expansion microscopy, high‐throughput serial electron microscopy followed by single‐profile tracking, and subsequent connectome analyses represents the most promising future avenues for this.

## Author Contributions

Claudia Groh, Wolfgang Rössler, and Andrea Rafaela Nicolaidou designed the study. Basil el Jundi and Andrea Rafaela Nicolaidou established the tracer injection setup. Andrea Rafaela Nicolaidou performed neuronal tracing, antibody labeling, immunohistochemistry, and confocal imaging. Basil el Jundi developed the framework for the 3D reconstructions. Andrea Rafaela Nicolaidou did the data analyses and created the 3D reconstructions from confocal images and *Z*‐stacks. Claudia Groh, Basil el Jundi, Wolfgang Rössler, and Andrea Rafaela Nicolaidou discussed the data analyses and 3D reconstructions. Claudia Groh and Wolfgang Rössler obtained funding. Claudia Groh and Andrea Rafaela Nicolaidou provided the first manuscript draft. All authors contributed to revisions and approved the manuscript.

## Funding

The work was financially supported by DFG grants #430253184 (to C.G.) and #466488864 and #272768235 (to W.R.).

## Conflicts of Interest

The authors declare no conflicts of interest.

## Data Availability

All relevant data are included in the manuscript. Raw data can be made available by the author upon reasonable request.
